# The Putative APSES Transcription Factor RgdA Governs Growth, Development, Toxigenesis, and Virulence in Aspergillus fumigatus

**DOI:** 10.1128/mSphere.00998-20

**Published:** 2020-11-11

**Authors:** Sang-Cheol Jun, Yong-Ho Choi, Min-Woo Lee, Jae-Hyuk Yu, Kwang-Soo Shin

**Affiliations:** a Department of Microbiology, Graduate School, Daejeon University, Daejeon, Republic of Korea; b Soonchunhyang Institute of Medi-bio Science, Soonchunhyang University, Cheonan, Republic of Korea; c Department of Bacteriology, University of Wisconsin–Madison, Madison, Wisconsin, USA; d Department of Systems Biotechnology, Konkuk University, Seoul, Republic of Korea; University of Georgia

**Keywords:** APSES transcription factor, *Aspergillus fumigatus*, RgdA, gliotoxin, transcriptomics, virulence

## Abstract

The APSES transcription factor (TF) in *Aspergillus* species is known to govern diverse cellular processes, including growth, development, and secondary metabolism. Here, we investigated functions of the *rgdA* gene (Afu3g13920) encoding a putative APSES TF in the opportunistic human-pathogenic fungus Aspergillus fumigatus. The *rgdA* deletion resulted in significantly decreased hyphal growth and asexual sporulation. Consistently, transcript levels of the key asexual developmental regulators *abaA*, *brlA*, and *wetA* were decreased in the Δ*rgdA* mutant compared to those in the wild type (WT). Moreover, Δ*rgdA* resulted in reduced spore germination rates and elevated transcript levels of genes associated with conidium dormancy. The conidial cell wall hydrophobicity and architecture were changed, and levels of the RodA protein were decreased in the Δ*rgdA* mutant. Comparative transcriptomic analyses revealed that the Δ*rgdA* mutant showed higher mRNA levels of gliotoxin (GT)-biosynthetic genes and GT production. While the Δ*rgdA* mutant exhibited elevated production of GT, Δ*rgdA* strains showed reduced virulence in the mouse model. In addition, mRNA levels of genes associated with the cyclic AMP (cAMP)-protein kinase A (PKA) signaling pathway and the SakA mitogen-activated protein (MAP) kinase pathway were increased in the Δ*rgdA* mutant. In summary, RgdA plays multiple roles in governing growth, development, GT production, and virulence which may involve attenuation of PKA and SakA signaling.

**IMPORTANCE** Immunocompromised patients are susceptible to infections with the opportunistic human-pathogenic fungus Aspergillus fumigatus. This fungus causes systemic infections such as invasive aspergillosis (IA), which is one of the most life-threatening fungal diseases. To control this serious disease, it is critical to identify new antifungal drug targets. In fungi, the transcriptional regulatory proteins of the APSES family play crucial roles in controlling various biological processes, including mating, asexual sporulation and dimorphic growth, and virulence traits. This study found that a putative APSES transcription factor, RgdA, regulates normal growth, asexual development, conidium germination, spore wall architecture and hydrophobicity, toxin production, and virulence in A. fumigatus. Better understanding the molecular mechanisms of RgdA in human-pathogenic fungi may reveal a novel antifungal target for future drug development.

## Abstract (precis)

Immunocompromised patients are susceptible to infections with the opportunistic human-pathogenic fungus Aspergillus fumigatus. This fungus causes systemic infections such as invasive aspergillosis (IA), which is one of the most life-threatening fungal diseases. To control this serious disease, it is critical to identify new antifungal drug targets. In fungi, the transcriptional regulatory proteins of the APSES family play crucial roles in controlling various biological processes, including mating, asexual sporulation and dimorphic growth, and virulence traits. This study found that a putative APSES transcription factor, RgdA, regulates normal growth, asexual development, conidium germination, spore wall architecture and hydrophobicity, toxin production, and virulence in A. fumigatus. Better understanding the molecular mechanisms of RgdA in human-pathogenic fungi may reveal a novel antifungal target for future drug development.

## INTRODUCTION

The APSES (Asm1p, Phd1p, Sok2p, Efg1p, and StuAp) family transcription factors (TFs) are conserved from yeasts to plants and are known to govern growth, morphogenesis, development, and secondary metabolism in the kingdom Fungi (1). Their biological roles have been well studied in the budding yeast Saccharomyces cerevisiae (2–4). For instance, the Swi4 and Swi6 proteins of S. cerevisiae have been demonstrated to function as a Swi4-, Swi6-dependent cell cycle box binding factor (SBF) complex (5). This heteromeric SBF complex functions with a DNA-binding subunit of the Swi4 protein and a regulatory subunit of the Swi6 protein, and it extensively regulates the G_1_/S cell cycle control transcriptional program (6, 7). Both proteins in the SBF complex have an ankyrin repeat domain that mediates protein-protein interactions, and only the Swi4 protein has an APSES-type DNA-binding domain belonging to the basic helix-loop-helix (bHLH) class of TF. In late G_1_ phase, two transcription complexes, the MluI cell cycle box binding factor (MBF) complex and the SBF complex, are activated by the G_1_ cyclin Cln3-Cdc28 protein kinase complex. The Swi6 protein is a critical component of the SBF and MBF, and the complexes bind to Swi4 and Mbp1, respectively. The Mbp1, too, is an APSES-type HLH DNA-binding protein that forms the MBF complex with Swi6. These regulatory complexes are known to induce the gradational expression of several genes involved in mitotic cell division and DNA synthesis (8, 9).

In contrast, functional studies of the APSES TFs in *Aspergillus* are somewhat limited. The most studied of them is StuA, an APSES-type factor that is involved in asexual sporulation and whose expression is cell type specific and dependent on BrlA. StuA affects spatial localization of the AbaA protein by precisely controlling *abaA* expression during conidiophore morphogenesis (10, 11). An APSES transcription factor, RgdA (the Swi4p homolog in Aspergillus nidulans), controls differentiation of phialide in A. nidulans. An *rgdA* deletion mutant showed an aborting conidial head, a reduced number of conidia, and retarded growth (12). During the asexual developmental stage, the RgdA protein also positively regulates expression of the *brlA* and *abaA* genes. These phenotypes were partially suppressed by the *veA1* mutation (12). Two APSES proteins, AfRafA and AfStuA, have been identified in Aspergillus flavus. AfRafA controls expression of the aflatoxin (AF) biosynthetic gene cluster, AF production, and development of conidia and sclerotia. AfStuA regulates biosynthesis of AF in an AflR-dependent manner (13). Collectively, these APSES TFs in A. flavus regulate fungal development, toxin production, and pathogenicity (13).

In the human opportunistic pathogen Aspergillus fumigatus, only the StuA-like APSES protein has been studied. Loss of *stuA* resulted in impaired asexual reproduction, abnormal conidiophore formation, and precocious germination (14). In addition, StuA played a key role in regulating secondary metabolism in A. fumigatus (14, 15). In this study, we investigated functions of another APSES protein, RgdA, in A. fumigatus, and we report that RgdA governs growth, asexual development, cell wall hydrophobicity, gliotoxin production, virulence, and signal transduction pathways in A. fumigatus.

## RESULTS

### Summary of A. fumigatus RgdA.

**FIG 1:**
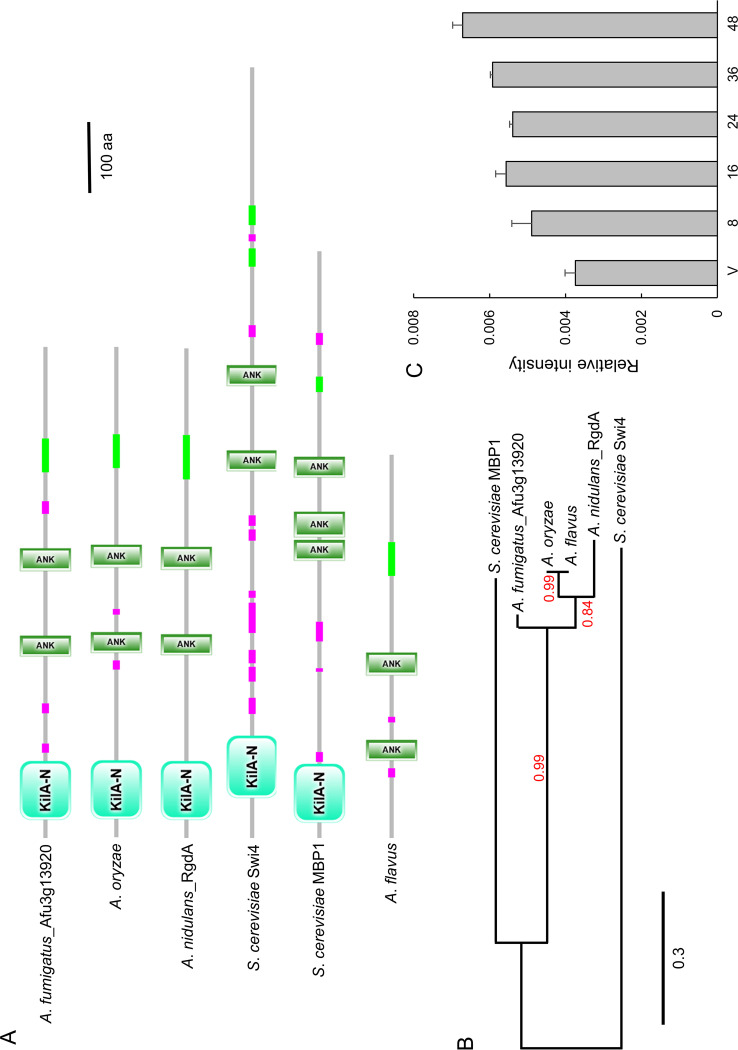
Summary of RgdA in A. fumigatus. (A) Schematic presentation of the domain architecture of the RgdA orthologues using SMART (http://smart.embl-heidelberg.de). (B) Phylogenetic tree of the RgdA-like proteins in various fungi, constructed based on the matrix of pairwise distances between the sequences. (C) Levels of *rgdA* mRNA during the life cycle of A. fumigatus WT.

The open reading frame (ORF) of *rgdA* in A. fumigatus Af293 (Afu3g13920) consists of 2,094 bp with 2 introns, predicted to encode a 697-amino-acid length protein. As shown in Fig. 1A, the domain architecture of A. fumigatus RgdA contains an APSES-type DNA binding domain in the N-terminal region and two ankyrin repeat-containing domains. These two domains are conserved in all *Aspergillus* RgdA-like proteins except in A. flavus, where the N-terminal region of the KilA-N domain is truncated. RgdA of A. fumigatus is an orthologue of S. cerevisiae MBP1 and Swi4, with amino acid sequence identities of 32.6% and 24.1%, respectively. On the other hand, it shows 75.6% amino acid sequence identity with RgdA of A. nidulans and 77.9% to 79.4% identity with RgdA homologs in Aspergillus oryzae and A. flavus. In an unrooted phylogenetic analysis based on the amino acid sequence, RgdA homologs of *Aspergillus* are clustered in the same group (Fig. 1B). We examined levels of the *rgdA* mRNA throughout the life cycle and found that they were low during the early developmental phase and increased in later phases of development (Fig. 1C).

### RgdA affects mycelial growth and asexual sporulation.

**FIG 2:**
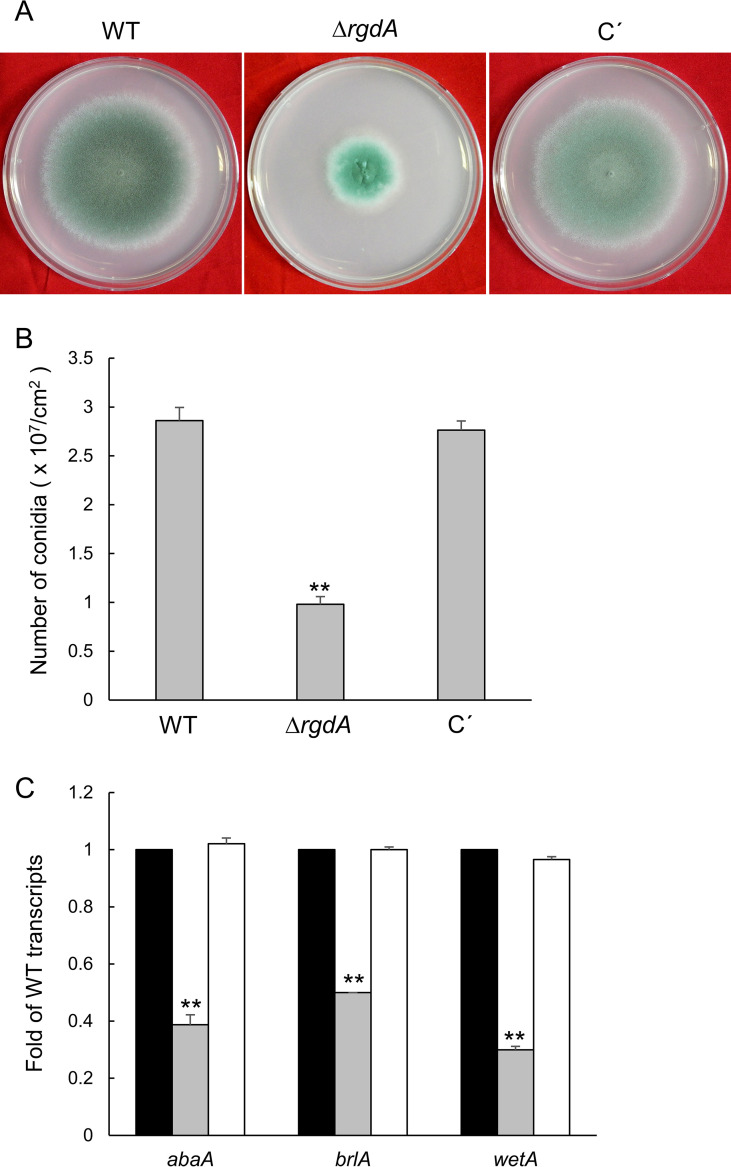
RgdA is required for proper fungal growth and development. (A) Colonies of WT (AF293), Δ*rgdA*, and complemented (C′) strains point-inoculated on solid MMY and grown for 4 days. (B) Numbers of conidia produced by each strain per growth area. (C) mRNA levels of the key asexual developmental regulators in the Δ*rgdA* mutant relative to the WT at 24 h determined by qRT-PCR. Fungal cultures were done in liquid MMY, and mRNA levels were normalized using the *ef1α* gene. Data are means and standard deviations from three independent experiments. **, *P* < 0.01 (analysis of variance [ANOVA]).

To investigate functions of *rgdA*, we generated the Δ*rgdA* null mutant and complemented strains. The Δ*rgdA* mutant showed significantly reduced radial growth compared to wild-type (WT) and complemented strains (Fig. 2A). Quantitative analyses of numbers of conidia per plate grown on solid medium further demonstrated that asexual spore production in the Δ*rgdA* mutant (0.98 × 10^7^ conidia/cm^2^) was dramatically decreased to about 35% of that in WT and complemented strains (Fig. 2B). In accordance with this observation, in the Δ*rgdA* mutant, mRNA levels of the key asexual developmental regulators *abaA*, *brlA*, and *wetA* were significantly reduced (Fig. 2C). These results suggest that RgdA is necessary for both normal growth and proper conidiation.

### RgdA may attenuate cAMP-dependent PKA signaling.

**FIG 3:**
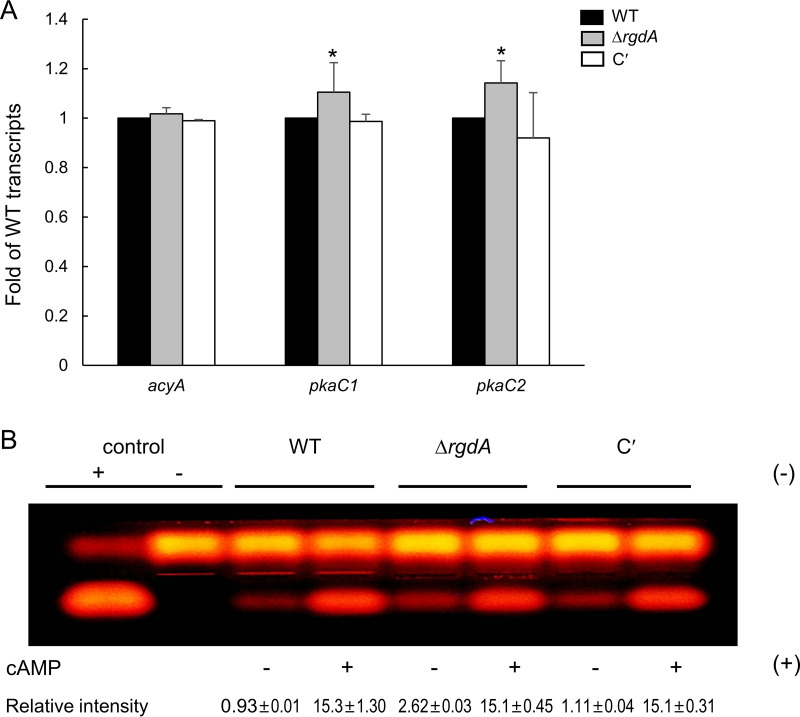
RgdA negatively affects the cAMP-PKA signaling pathway. (A) Expression levels of *acyA*, *pkaC1*, and *pkaC2* mRNA in WT, Δ*rgdA*, and complemented (C′) strains analyzed by qRT-PCR. Statistical differences between strains were evaluated with ANOVA. *, *P* < 0.05. (B) PKA activity levels of three strains as monitored by gel electrophoresis. Each strain was grown in MMY for 3 days at 37°C, and mycelial extract was analyzed.

To begin to investigate a possible relationship between RgdA and the cyclic AMP (cAMP)-protein kinase A (PKA) signaling pathway, we analyzed mRNA levels of selected PKA pathway-related genes. As shown in Fig. 3A, *pkaC1* and *pkaC2* mRNA levels were significantly higher in the Δ*rgdA* mutant than in WT strain. To investigate this further, we assessed PKA activity using the peptide substrate kemptide. While all tested strains exhibited similar levels of PKA activity in the presence of cAMP, the Δ*rgdA* mutant showed about 2-fold-higher PKA activity in the absence of cAMP (Fig. 3B). These results indicate that RgdA is required for properly controlling expression of *pkaC1* and *pkaC2* and PKA activity, suggesting that RgdA may negatively regulate a cAMP-PKA signaling pathway.

### RgdA is necessary for proper germination of conidia.

**FIG 4:**
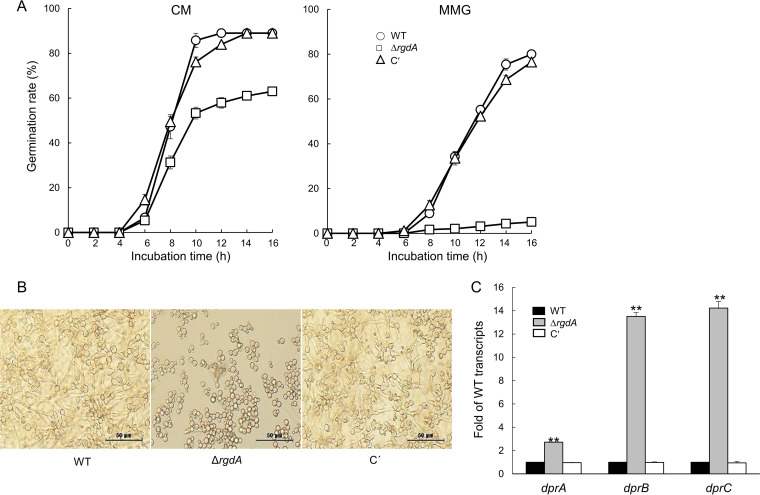
RgdA is necessary for proper spore germination. (A) Kinetics of germ tube outgrowth in A. fumigatus strains inoculated in liquid CM and MMG at 37°C The number of conidia showing germ tube protrusion was recorded at 2-h intervals and is presented as a percentage of the total number of conidia in each microscope field. CM, complete medium; MMG, glucose minimal medium; C′, complemented strain. (B) Germinated spores. Conidia were inoculated in MMG and incubated at 37°C for 14 h. (C) Effect of *rgdA* on conidial germination-regulatory genes. RNA was extracted from 24-h cultures of each strain. Strains were incubated in MMY medium at 37°C, and mRNA levels were normalized using the *ef1α* gene. Data are means and standard deviations from three independent experiments. **, *P* < 0.01 (ANOVA).

To investigate a potential role of RgdA in controlling spore germination, we analyzed the kinetics of germ tube emergence in the Δ*rgdA* conidia in comparison to those of WT and complemented strains in triplicate. As shown in Fig. 4A, germination rate of the Δ*rgdA* conidia was remarkably reduced compared to that of other strains. Germination rate of the Δ*rgdA* conidia in complete medium (CM) was 30% lower than that of WT and complemented strains. Moreover, while about 80% of WT and complemented strains’ conidia germinated, only 5% of the Δ*rgdA* spores germinated on minimal medium containing glucose (MMG) after 16 h of incubation (Fig. 4A and B). We further investigated mRNA levels of the dehydrin-like-protein-encoding genes *dprA*, *dprB*, and *dprC*, which negatively affect conidial dormancy. As shown in Fig. 4C, mRNA levels of *dprA* in the Δ*rgdA* mutant were about 3 times higher than those in WT and complemented strains, and mRNA levels of *dprB* and *dprC* were increased about 14 times in the Δ*rgdA* mutant compared to those in WT and complemented strains. These results suggest that RgdA regulates spore germination likely through negative regulation of the *dprA*, *dprB*, and *dprC* genes.

### RgdA negatively regulates the SakA MAP kinase pathway.

**FIG 5:**
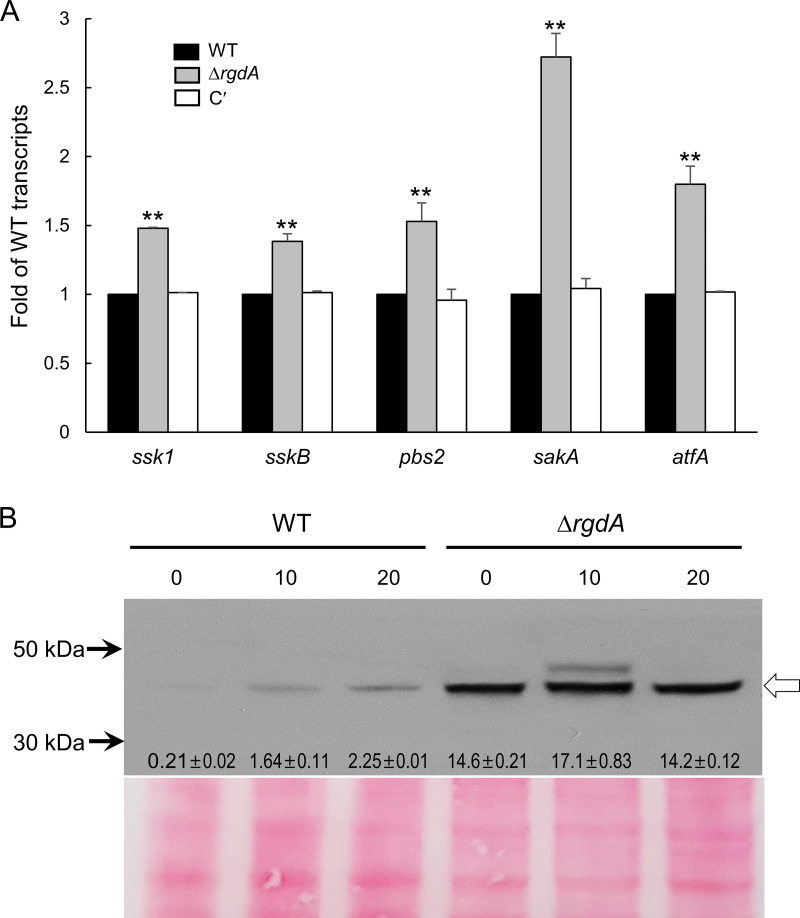
RgdA negatively regulates the SakA MPKA pathway. (A) mRNA expression levels of SakA MAP kinase pathway-related genes in WT, Δ*rgdA*, and complemented (C′) strains analyzed by qRT-PCR. Statistical differences between strains were evaluated with ANOVA. **, *P* < 0.01. (B) Conidia of the indicated strains were grown for 14 h in MMY and treated with 10 mM H_2_O_2_. Aliquots of cells were harvested at the indicated times and used to prepare total protein extracts. Protein extracts were analyzed by immunoblotting with anti-phospho-p38 antibody. The arrow indicates phospho-p38 protein. Relative intensity values are presented.

A previous study demonstrated that the dehydrin-like proteins DprA and DprB act downstream of the SakA MAPK cascade and that SakA interacted with AtfA (16). To examine whether RgdA-mediated repression of the *dpr* genes occurs via controlling the SakA MAPK pathway, we analyzed transcript levels of SakA MAPK pathway-related genes and phosphorylation levels of SakA. Levels of mRNA of all genes examined were increased about 1.5- to 2.5-fold in the Δ*rgdA* mutant (Fig. 5A). We then carried out anti-phospho-p38 immunoblotting with total soluble protein extracts from WT and Δ*rgdA* strains treated with 10 mM H_2_O_2_ for 10 and 20 min. A protein with the predicted molecular mass of SakA became transiently phosphorylated in response to the oxidative stress. However, the phosphorylation levels of the Δ*rgdA* strain were much higher than those in the WT strain, even in the absence of oxidative stress (0 min) (Fig. 5B). These results indicate that RgdA negatively regulates the SakA MAPK pathway, which in turn leads to downregulation of the *dpr* genes.

### Loss of *rgdA* changes the conidial hydrophobicity and conidial wall architecture.

**FIG 6:**
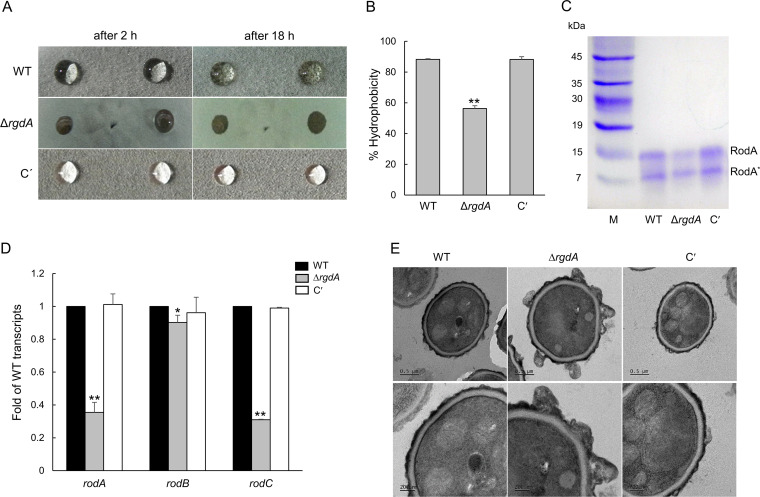
RgdA is needed for proper conidial hydrophobicity and cell wall architecture. (A) Hydrophobicity test. Strains were cultured on MMY agar plates for 4 days at 37°C, and 10 μl of a detergent solution (0.2% SDS, 50 mM EDTA) was applied dropwise onto the surface of a colony. The droplets were observed for penetration into the colonies. C′, complemented strain. (B) Percent hydrophobicity of three strains by the MATS test. (C) SDS-PAGE analysis of the hydrophobin RodA of relevant strains. RodA was extracted from the dried conidia (10^9^) with hydrofluoric acid (HF). RodA*, degraded form of RodA due to HF treatment. (D) qRT-PCR analysis of hydrophobin genes in WT, Δ*rgdA*, and complemented strains. Statistical differences between strains were evaluated with ANOVA. **, *P* < 0.01; *, *P* < 0.05. (E) Transmission electron micrographs of conidia. The conidia were harvested from the cells cultured on MMY for 5 days, and ultrathin specimens were prepared for transmission electron microscopy.

The hydrophobicity of conidia is one of the key factors determining the virulence of fungal pathogens, and the physicochemical properties of hydrophobin affect conidial hydrophobicity. To understand the role of RgdA in governing fungal hydrophobicity, we analyzed hydrophobicity of WT, Δ*rgdA*, and complemented colonies using a detergent permeation assay. As shown in Fig. 6A, the detergent solution began to permeate the colony after 2 h and completely penetrated it at 18 h in the Δ*rgdA* mutant. In contrast, WT and complemented strains still displayed detergent solution droplets on the colony surface at 18 h (Fig. 6A). To further confirm the hydrophobicity of conidia, we performed a MATS (microbial adhesion to solvents) test. Hydrophobicity of the Δ*rgdA* conidia was about 30% lower than that of WT and complemented conidia (Fig. 6B). Then we extracted the conidial hydrophobin RodA from the conidia and analyzed it by SDS-PAGE. As shown in Fig. 6C, the amount of the RodA protein was reduced by the loss of *rgdA*. Levels of mRNA of the hydrophobin genes *rodA*, *rodB*, and *rodC* were also significantly decreased in the Δ*rgdA* mutant (Fig. 6D). These results suggest that RgdA positively regulates expression of the *rod* genes and subsequently confers proper conidial hydrophobicity. To characterize the link between the change of conidial hydrophobicity and conidia cell wall structure, we observed the fine structure of the conidia of the three strains by using transmission electron microscopy (TEM). As shown in Fig. 6E, the structure of the Δ*rgdA* conidia surface was very different from that of WT and complemented conidia. Although their relevance is not clear, several irregular protrusions were observed on the surfaces of Δ*rgdA* conidia (Fig. 6E), suggesting that RgdA is necessary for proper conidial wall architecture.

### Transcriptomic analysis reveals that RgdA contributes to gliotoxin biosynthesis.

**FIG 7:**
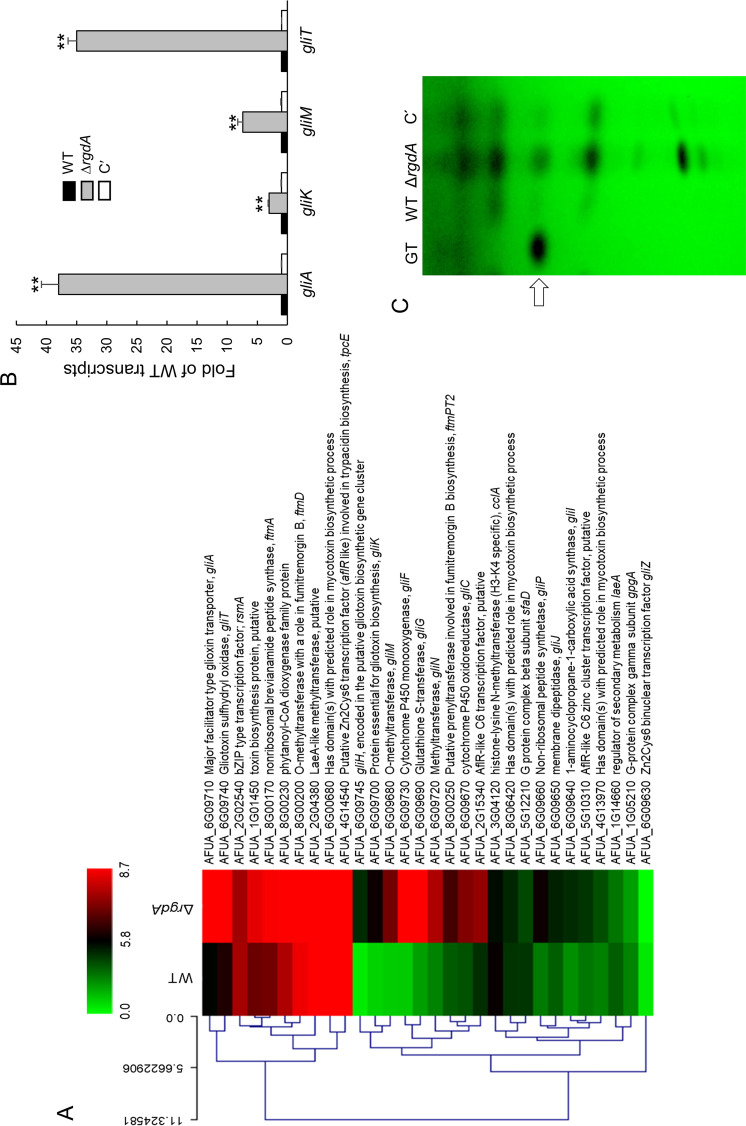
RgdA downregulates GT production. (A) Heat map of genes encoding toxin-related proteins. Most gliotoxin biosynthetic genes were upregulated by the loss of *rgdA*. (B) qRT-PCR analysis of GT-related genes in WT, Δ*rgdA*, and complemented (C′) strains. Statistical differences between WT and mutant strains were evaluated with ANOVA. **, *P* < 0.01. (C) Determination of GT production in WT, Δ*rgdA*, and complemented strains. The culture supernatant of each strain was extracted with chloroform and subjected to TLC. The arrow indicates GT.

To further characterize the complex role of RgdA, we performed RNA-Seq analysis using Δ*rgdA* and WT strains. Total RNAs were isolated from 24-h-old cultures of WT and Δ*rgdA* strains in MMG with 0.1% yeast extract (MMY). Of 9,859 annotated genes of A. fumigatus, 2,261 genes were differentially expressed more than 2-fold (*P* < 0.05), of which 1,227 genes were upregulated and 1,034 genes were downregulated (Fig. S1A). The functional category of the genes was analyzed by using the gene ontology (GO) terms that were enriched in differentially expressed genes (DEGs). The top significant molecular function GO category is “lipase activity,” and the top significant cellular component GO categories are “vacuole,” “cell wall,” and “extracellular region.” The top significant biological process GO categories are “mycotoxin biosynthetic process,” “pathogenesis,” and “germination” (Fig. S1B). The top 20 DEGs with elevated mRNA levels in the Δ*rgdA* strain compared to the WT are listed in Table S1. Notably, mRNA levels of the gliotoxin (GT) biosynthesis genes *gliA* (encoding the MFS [major facilitator superfamily] GT efflux transporter) and *gliF* (encoding cytochrome P450 oxidoreductase) were over 200-fold higher in the Δ*rgdA* mutant than WT. Most of the downregulated genes were those encoding polysaccharide-degrading enzymes (Table S2). As shown in Fig. 7A, most of the GT biosynthetic clustered genes were upregulated by Δ*rgdA*. To corroborate the transcriptome sequencing (RNA-Seq) results, we examined mRNA levels of four *gli* genes by quantitative reverse transcription-PCR (qRT-PCR). Levels of *gliA*, *gliK*, *gliM*, and *gliT* transcripts were significantly higher (3- to 37-fold) in the Δ*rgdA* strain than in the WT and complemented strains (Fig. 7B). We also examined the effect of Δ*rgdA* in GT production. We assessed levels of GT in WT, Δ*rgdA*, and complemented strains and found that the Δ*rgdA* mutant produced about 5-fold more GT and other secondary metabolites than the WT and complemented strains (Fig. 7C).

10.1128/mSphere.00998-20.1 FIG S1 RNA-Seq analysis of WT and Δ*rgdA* strains. (Left) Volcano plot of the RNA-Seq data sets for comparison of the differentially expressed genes. Red and green points mark the genes with significantly increased or decreased expression in the Δ*rgdA* versus the WT strain, respectively (*P* < 0.05). (Right) Functional categories of DEGs. Green bars represent genes whose mRNA levels decreased in the Δ*rgdA* strain, whereas red represents genes whose mRNA levels increased in the Δ*rgdA* strain more than 2-fold (*P* < 0.05). Download FIG S1, PDF file, 0.2 MB. Copyright © 2020 Jun et al. 2020 Jun et al. This content is distributed under the terms of the Creative Commons Attribution 4.0 International license.

10.1128/mSphere.00998-20.2 TABLE S1 Top 20 upregulated genes in the Δ*rgdA* strain relative to the WT strain (*P* < 0.01). Download Table S1, DOCX file, 0.01 MB. Copyright © 2020 Jun et al. 2020 Jun et al. This content is distributed under the terms of the Creative Commons Attribution 4.0 International license.

10.1128/mSphere.00998-20.3 TABLE S2 Top 20 downregulated genes in the Δ*rgdA* strain relative to the WT strain (*P* < 0.01). Download Table S2, DOCX file, 0.01 MB. Copyright © 2020 Jun et al. 2020 Jun et al. This content is distributed under the terms of the Creative Commons Attribution 4.0 International license.

### RgdA plays an important role in virulence.

**FIG 8:**
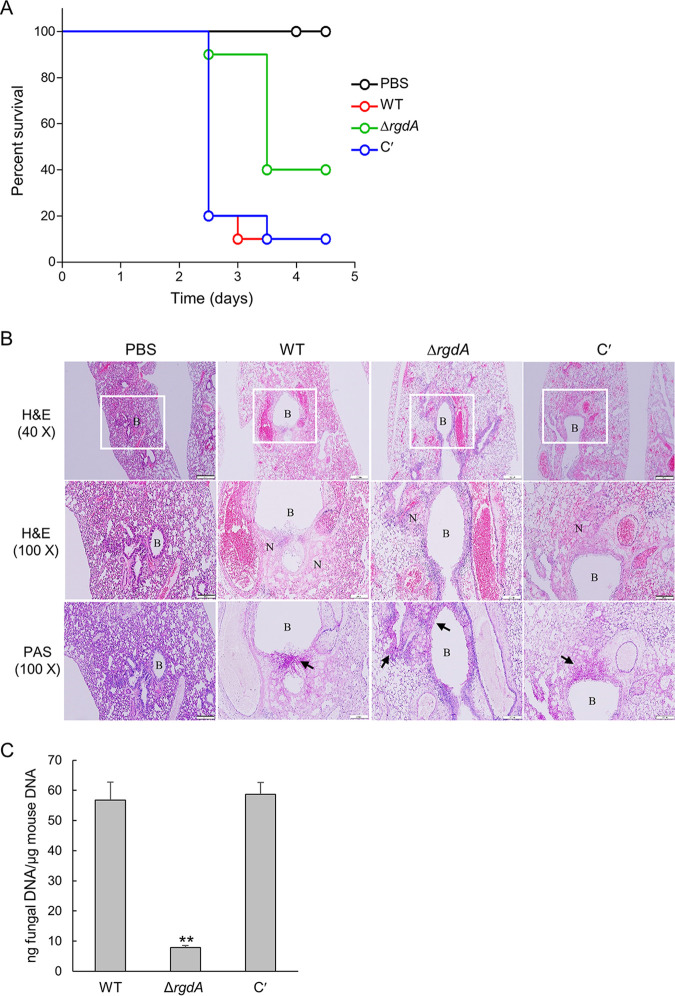
A role of RgdA in virulence. (A) Survival curve of mice infected with WT, Δ*rgdA*, and complemented (C′) strains (*n* = 10/group). (B) Lung sections were stained with H&E or PAS. B, bronchiole; N, necrosis. The arrow indicates fungal mycelium. Bars, 200 μm (40×) and 100 μm (100×). (C) Fungal burden in the lungs of mice infected with WT, Δ*rgdA*, and complemented strains. Data are means and standard deviations from three independent experiments. **, *P* < 0.01 (ANOVA).

In order to investigate the pathological significance of the RgdA protein during A. fumigatus infection, conidia of WT, Δ*rgdA*, and complemented strains were intranasally introduced into neutropenic mice, which were generated by combinatorial administrations of cyclophosphamide and cortisone acetate. Pathological outcomes were monitored by assessing mouse survival. In the survival curve analysis, while a group infected with the WT strain showed the first mortality at 2.5 days after infection and displayed a 10% survival rate within 3.5 days, a group infected with the Δ*rgdA* mutant showed the first death at 2.5 days and a 40% survival rate even after 5 days (*P = *0.0409). Complementation of the Δ*rgdA* mutation with the WT allele restored the virulence to the WT level (Fig. 8A). Next, to understand the basis for the observed differences in mouse survival, lung tissue sections were prepared from Δ*rgdA* and complemented strain-infected mice after 3 days of inoculation and stained with hematoxylin and eosin (H&E) or periodic acid-Schiff (PAS) to compare the extent of fungal impact and hyphal growth. H&E staining showed that infection led to lung damage. However, compared to the WT, which caused severe necrosis around bronchiole regions and disruption of alveolar structure and bronchial wall, the Δ*rgdA* mutant led to milder necrosis and disruption of bronchi structure. Furthermore, as observed with PAS staining, milder damage caused by the Δ*rgdA* strain infection was associated with reduced hyphal growth and infiltration into peribronchial regions. Conidia of the complemented strain resulted in severe tissue damage and hyphal growth comparable to WT levels (Fig. 8B). In addition, loss of *rgdA* significantly decreased (about 5-fold) the pulmonary fungal burden of mice (Fig. 8C).

## DISCUSSION

RgdA of A. fumigatus is a homolog of the Mbp1 protein of the budding yeast S. cerevisiae, a putative APSES TF. APSES TFs function as key regulators of fungal morphogenesis and development present in *Aspergillus* genomes, but only the *stuA* gene has been studied in A. fumigatus (10, 12, 14, 15, 17). The deletion of *rgsA* resulted in decreased mycelial growth and conidiation and reduced mRNA levels of key asexual development regulators compared to WT (Fig. 2). The mRNA levels of PKA signaling components were higher in the Δ*rgdA* strain than in WΤ and complemented strains. Furthermore, the Δ*rgdA* strain showed higher PKA activity in the absence of cAMP (Fig. 3), suggesting the presence of free PKA catalytic subunits in the cytoplasm (18). Based on these observations, we can propose that RgdA of A. fumigatus is necessary for normal growth and proper asexual development, which may involve regulation of the cAMP-PKA signaling pathway.

The Dpr proteins are known to be activated by the stress-activated kinase SakA MAPK pathway and function as chaperone molecules responding to osmotic, oxidative, pH, and cold stress independently, and they endure stress during the conidial dormancy period (16, 19). To the best of our knowledge, this is the first study revealing that RgdA negatively controls expression of dehydrin-like *dpr* genes and SakA MAPK pathway-related genes (Fig. 4 and 5) and that the absence of RgdA leads to hyperphosphorylation of SakA (Fig. 5). These results lead us to speculate that RgdA downregulates expression of the *dpr* genes by attenuating the SakA MAPK pathway.

RgdA is also required for the proper conidia hydrophobicity, conidia cell wall architecture, and synthesis of the hydrophobin RodA protein (Fig. 6). The cell wall of the infective fungal conidium is covered with a melanin layer and the rodlet-shaped outer layer, an amyloid fiber composed of the hydrophobin RodA. The ability of airborne conidia to reach alveoli is primarily dependent on the hydrophobic rodlet layer, which promotes the dispersion of spores (20). In addition, the rodlet layer formed by RodA interferes with the recognition of spores by the human immune system (21). Recent studies have shown that only RodA is involved in the hydrophobicity, formation of rodlets, physical resistance, and immunological inertia of conidia (22). According to our results, formation of the proper rodlet layer requires RgdA, and the absence of *rgdA* would likely cause defects in fungal pathogenicity.

The well-studied APSES TF StuA has been shown to positively regulate the aflatoxin biosynthetic cluster genes in A. flavus and several secondary metabolite biosynthetic cluster genes in A. fumigatus (13, 15). In contrast, our studies demonstrate that RgdA in A. fumigatus represses expression of the GT gene clusters and production of GT (Fig. 7). GT is a major potent toxin and an important virulence factor of A. fumigatus (23). Nonetheless, the Δ*rgdA* mutant produced a significantly larger amount of GT than the WT and complemented strains, and the virulence was greatly reduced by Δ*rgdA* (Fig. 8). Our histological and fungal burden studies with infected mice showed that the deletion of *rgdA* resulted in milder necrosis and disruption of the bronchiole region than was seen with WT and complemented strains. Furthermore, Δ*rgdA* significantly decreased (about 5-fold) the pulmonary fungal burden of mice (Fig. 8). Collectively, these results show that despite the elevated levels of GT, the absence of *rgdA* leads to reduced virulence, which may be associated with the lowered conidial hydrophobicity and RodA.

**FIG 9:**
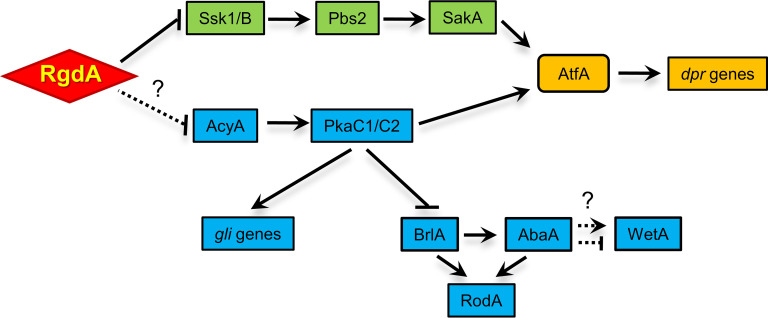
Genetic model depicting the role of RgdA in A. fumigatus. RgdA negatively regulates the cAMP-PKA signaling and SakA MAP kinase pathway. The lack of RgdA leads to the enhanced activation of the cAMP-PKA signaling pathway and SakA MAP kinase pathway, which in turn leads to increased expression of *gli* genes, *dpr* genes, and *rod* genes.

In summary, we propose a genetic model depicting the complex regulatory role of RgdA in A. fumigatus (Fig. 9). In this model, RgdA negatively acts at or upstream of the cAMP-PKA and stress-activated SakA MAPK signaling pathways, which activate GT biosynthesis and expression of the *dpr* genes, through PkaC1/C2 and AtfA, respectively. Unlike their stimulating action on GT production, PkaC1/C2 repress asexual developmental activators and the subsequent RodA production. Further studies are needed to identify downstream direct targets and potential upstream regulators of RgdA in A. fumigatus.

## MATERIALS AND METHODS

### Ethics statement.

All of the animal procedures in this study were reviewed and approved by the Institutional Animal Care and Use Committee of Daejeon University (DJUARB2019-024).

### Strains and culture conditions.

A. fumigatus AF293.1 (*AfpyrG1*) was used to generate the Δ*rgdA* mutant, and AF293 was used as the wild type (WT). Fungal strains were grown on glucose minimal medium (MMG) or MMG with 0.1% yeast extract (MMY) with appropriate supplements as described previously (24). Liquid submerged culture and phenotypic analyses on air-exposed culture were performed as described previously (25). To examine secondary metabolite production, conidia of relevant strains were inoculated in 5 ml of liquid MMY and incubated at 37°C for 7 days.

### Generation of the Δ*rgdA* mutant in A. fumigatus.

The deletion construct generated employing double-joint PCR (26) containing the A. nidulans selective marker (*AnipyrG*) with the 5′ and 3′ flanking regions of the A. fumigatus *rgdA* gene (Afu3g13920) was introduced into the recipient strains (27). The selective marker was amplified from A. nidulans FGSC A4 genomic DNA with the primer pair oligo697-oligo698. The null-mutant colonies were isolated and confirmed by diagnostic PCR (oligo378-oligo379), followed by restriction enzyme digestion. To complement the *rgdA*-null mutant, a single-joint PCR (SJ-PCR) method was used (26). The ORF of the *rgdA* gene with a promoter and a terminator was amplified with primer pairs where the 3′ reverse primer carries sequences overlapping the *hygB* gene’s 5′ end. Amplification of the *hygB* gene was carried out with primer pairs where the 5′ forward primer carries sequences overlapping *hygB* gene’s 3′ end. The final amplicon was amplified with the nested primer and introduced into the Δ*rgdA* strain. The oligonucleotides used in this study are listed in Table S3.

10.1128/mSphere.00998-20.4 TABLE S3 Oligonucleotides used in this study. Download Table S3, DOCX file, 0.02 MB. Copyright © 2020 Jun et al. 2020 Jun et al. This content is distributed under the terms of the Creative Commons Attribution 4.0 International license.

### Nucleic acid isolation and manipulation.

Total RNA isolation and quantitative RT-PCR (qRT-PCR) assays were performed as previously described (28–30). Expression of target gene mRNA was analyzed with appropriate oligonucleotide pairs (Table S3). For RNA-Seq analyses, 24-h-old cultures of WT and mutant strains were harvested from solid MMY. Total RNA was extracted and submitted to eBiogen, Inc. (Seoul, South Korea), for library preparation and sequencing.

### Measurement of germination rate and yield of conidia.

To examine conidial germination levels, conidia of WT and mutants were inoculated into 5 ml MMY broth at a concentration of 2 × 10^5^ conidia/ml and incubated at 37°C. Beginning two hours after inoculation, germination was assessed every 2 h. Three random visual fields were observed microscopically. The percent germination was calculated by the number of total conidia and germinated conidia in the visual field. For detection of conidia production levels, conidia were collected from an agar plug with 0.5% Tween 80 solution from the entire colony, filtered through Miracloth (Calbiochem, CA), and counted using a hemocytometer.

### Determination of conidial hydrophobicity.

To determine conidial hydrophobicity, three strains were cultured on MMY agar plates for 4 days at 37°C; 10 μl of a detergent solution (0.2% SDS, 50 mM EDTA) was applied dropwise onto the surface of a colony, and absorption patterns were observed. Hydrophobicity of the conidia was also assessed by aqueous-solvent partitioning assays, using the microbial adhesion to solvents (MATS) method (31). Briefly, 2 ml of conidial suspension in 0.1 M KNO_3_ (2 × 10^6^ to 7 × 10^6^ conidia/ml) were vortexed vigorously with 400 μl of hexadecane for 2 min. After separation of the two phases an aliquot of the aqueous phase was collected, and the number of conidia in the aqueous phase was determined using a hemocytometer. The percentage of conidia bound to solvent was calculated as (1 − *N*/*N*_0_) × 100, where *N*_0_ is the initial number of conidia in the aqueous phase and *N* is the residual number of conidia in the aqueous phase after partitioning. The RodA protein was extracted by incubating dry spores with hydrofluoric acid (HF) (10 μl per mg [dry weight]) for 72 h at 4°C (21). The contents were centrifuged at 10,000 rpm for 10 min, and the supernatants were subjected to acetone precipitation under cold condition. The obtained protein was reconstituted in Laemmli’s sample buffer, subjected to SDS-PAGE analysis, and visualized by Coomassie blue staining.

### Electron microscopy.

Conidia were fixed in 2.5% glutaraldehyde in 0.1 M phosphate, washed three times with 0.1 M phosphate, postfixed in 1% osmium tetroxide, incubated for 1 h in 0.1 M phosphate, and dehydrated for 15 min in a graded methanol series from 50% to 100%. Samples were embedded in Epon resin 812. The sections were examined with a Tecnai G2 Spirit Twin Bio-Transmission electron microscope (FEI, Hillsboro, OR, USA), with an accelerating voltage of 120 kV.

### Transcriptome analysis.

For control and test RNAs, the library was constructed using a QuantSeq 3′ mRNA-Seq library preparation kit (Lexogen, Inc., Austria) according to the manufacturer’s instructions. High-throughput sequencing was performed as single-end 75 sequencing using NextSeq 500 (Illumina, Inc., USA). QuantSeq 3′ mRNA-Seq reads were aligned using Bowtie2 (32). Bowtie2 indices were generated from either the genome assembly sequence or the representative transcript sequences for aligning to the genome and transcriptome. The alignment file was used for assembling transcripts, estimating their abundances, and detecting differential expression of genes. Differentially expressed genes were determined based on counts from unique and multiple alignments using coverage in Bedtools (33). The RT (read count) data were processed based on the quantile normalization method using EdgeR within R (R Development Core Team, 2016) using Bioconductor (34). Gene classification was based on searches in the DAVID (http://david.abcc.ncifcrf.gov/) and Medline (https://www.ncbi.nlm.nih.gov/) databases.

### Detection of GT.

The amount of GT was determined by the thin-layer chromatography (TLC) method as described previously (35). The cultured mycelial mass and the medium were mixed and used as the sample. The TLC silica plate was developed with toluene-chloroform (1:9, vol/vol).

### Murine virulence assay.

For the immunocompromised mouse model, we used outbred CrlOri:CD1 (ICR) (Orient Bio Inc, South Korea) female mice (30 g [body weight]; 6 to 8 weeks old), which were housed five per cage and had access to food and water *ad libitum*. Mice were immunosuppressed by treatment of cyclophosphamide (Cy; 250 mg/kg of body weight at days −3 and −1 and 125 mg/kg at day +1) and cortisone acetate (CA; 250 mg/kg at day −1 and 125 mg/kg at day +3). For conidium inoculation, mice were anesthetized with isoflurane and then intranasally infected with 1 × 10^7^ conidia of A. fumigatus strains (10 mice per fungal strain) in 30 μl of 0.01% Tween 80 in phosphate-buffered saline (PBS). Mice were monitored every 12 h for survival for 5 days after the challenge. Mock-infected mice included in all experiments were inoculated with sterile 0.01% Tween 80 in PBS. Mice were checked every 12 h for survival, and Kaplan-Meier survival curves were analyzed using the log-rank (Mantel-Cox) test for significance (*P* < 0.05).

### PKA assay and immunoblotting.

PKA activity was detected with a previously described method using a PepTag nonradioactive cAMP-dependent protein kinase assay kit (Promega, USA) (36). Total soluble proteins were extracted from WT and mutant strains subjected to 0, 10, and 20 min of 10 mM H_2_O_2_ treatment. Samples were separated using 12% SDS-PAGE and blotted onto nitrocellulose membrane. Blots were analyzed with anti-phospho-p38 MAPK (New England Biolabs, MA, USA) antibodies.

### Data availability.

The RNA-Seq data are available from the NCBI Gene Expression Omnibus (GEO) database (GSE123744).
