# Supplementary figures and images for: The Putative APSES Transcription Factor RgdA Governs Growth, Development, Toxigenesis, and Virulence in Aspergillus fumigatus
Source: mSphere. 2020 Nov 11;5(6):e00998-20. doi: 10.1128/mSphere.00998-20 (PMC7657592; doi:10.1128/mSphere.00998-20)

$\Delta$ rgdA vs WT

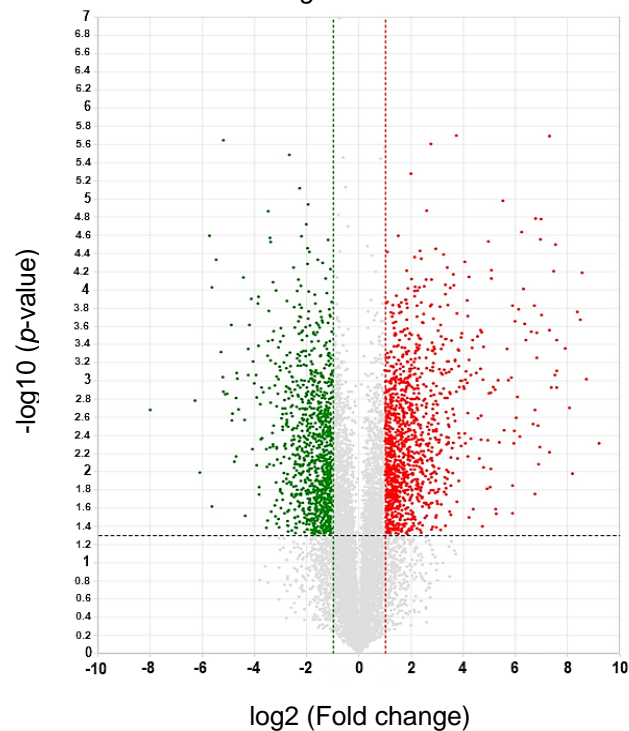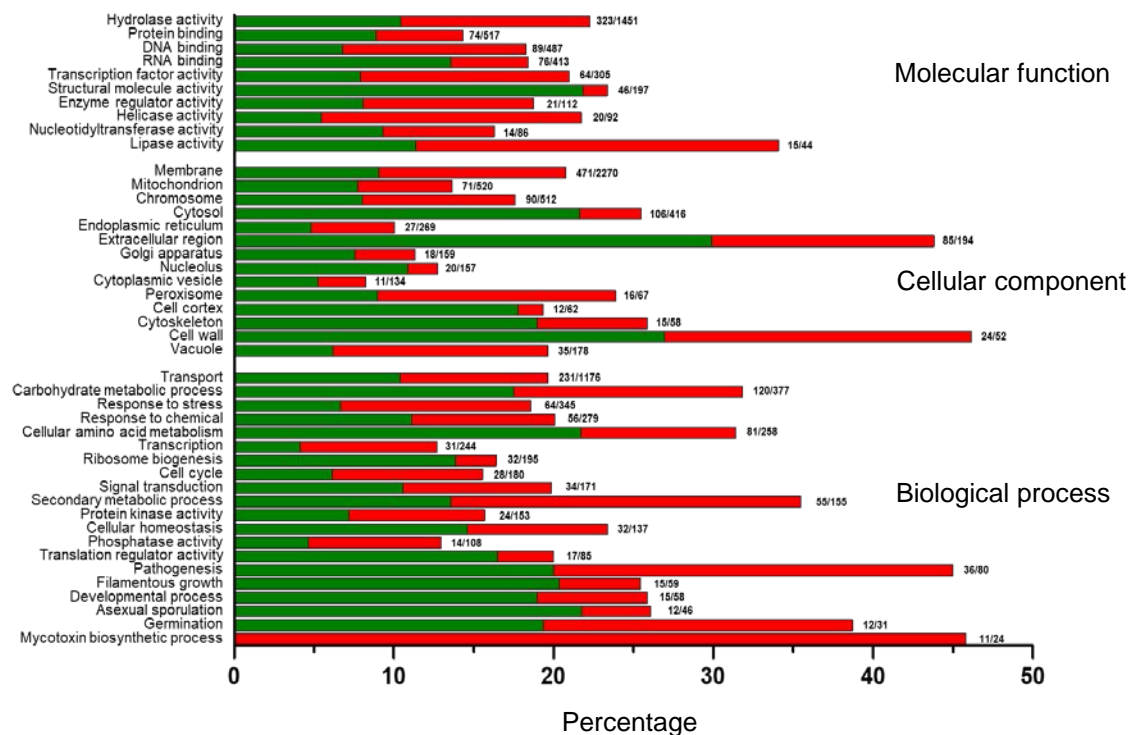

Supplement: FIG S1 [file mSphere.00998-20-sf001.pdf]
