# Supplementary material for: The Putative APSES Transcription Factor RgdA Governs Growth, Development, Toxigenesis, and Virulence in Aspergillus fumigatus
Source: mSphere. 2020 Nov 11;5(6):e00998-20. doi: 10.1128/mSphere.00998-20 (PMC7657592; doi:10.1128/mSphere.00998-20)
Supplement: TABLE S1 [file mSphere.00998-20-st001.docx]

**TABLE S1** Top 20 up-regulated genes in Δ*rgdA* relative to WT strain ( *p* < 0.01)

| Locus ID | Annotation | Fold change | *p*-value | Normalized RC |
| --- | --- | --- | --- | --- |
| AFUA_1G00990  AFUA_7G07100  AFUA_5G00300  AFUA_7G08400  AFUA_7G08620  AFUA_6G09710  AFUA_6G09730  AFUA_1G01040  AFUA_4G00830  AFUA_1G00930  AFUA_1G16000  AFUA_1G17750  AFUA_1G00840  AFUA_1G00210  AFUA_1G01000  AFUA_7G01780  AFUA_7G07130  AFUA_1G16050  AFUA_5G14740  AFUA_3G02640 | short chain dehydrogenase/reductase family protein  Pfs, NACHT and WD domain protein  zinc-binding oxidoreductase, putative  ankyrin repeat protein  hypothetical protein  MFS gliotoxin efflux transporter GliA  cytochrome P450 oxidoreductase GliF  Pfs domain protein  MFS peptide transporter, putative  hypothetical protein  serine/threonine protein kinase, putative  nicotinamide N-methyltransferase, putative  hypothetical protein  hypothetical protein  oxidoreductase, 2OG-Fe(II) oxygenase family  hypothetical protein  hypothetical protein  conserved serine-proline rich protein  fucose-specific lectin FleA  nucleoside-diphosphate-sugar epimerase family protein | 594.199  419.740  378.355  359.851  332.178  268.662  239.328  193.563  193.335  192.400  185.166  182.770  176.044  167.481  159.015  158.407  157.995  129.435  126.343  125.19 | 0.005  0.001  0.000  0.000  0.000  0.002  0.000  0.001  0.001  0.000  0.000  0.001  0.000  0.001  0.000  0.000  0.006  0.000  0.000  0.000 | 12.099  10.520  10.830  10.987  9.997  13.768  9.070  8.742  9.976  12.469  9.656  11.345  8.606  9.510  8.150  13.491  7.751  9.282  17.473  7.808 |
