# Supplementary material for: The Putative APSES Transcription Factor RgdA Governs Growth, Development, Toxigenesis, and Virulence in Aspergillus fumigatus
Source: mSphere. 2020 Nov 11;5(6):e00998-20. doi: 10.1128/mSphere.00998-20 (PMC7657592; doi:10.1128/mSphere.00998-20)
Supplement: TABLE S2 [file mSphere.00998-20-st002.docx]

**TABLE S2** Top 20 down-regulated genes in Δ*rgdA* relative to WT strain ( *p* < 0.01)

| Locus ID | Annotation | Fold change | *p*-value | Normalized RC |
| --- | --- | --- | --- | --- |
| AFUA_8G07080  AFUA_4G13750  AFUA_4G09480  AFUA_5G01990  AFUA_4G07850  AFUA_6G07070  AFUA_5G14240  AFUA_3G02720  AFUA_3G01910  AFUA_8G01850  AFUA_3G00320  AFUA_8G07060  AFUA_8G01640  AFUA_3G03060  AFUA_6G14540  AFUA_8G06430  AFUA_6G01830  AFUA_5G01030  AFUA_6G01800  AFUA_6G14510 | elastinolytic metalloproteinase Mep  penicillolysin/deuterolysin metalloprotease, putative  extracellular endo-1,4-beta-xylanase, putative  BYS1 domain protein, putative  endoglucanase, putative  cellobiohydrolase celD  ThiJ/PfpI family protein  MFS transporter, putative  cellobiohydrolase, putative  phosphate-repressible phosphate permease, putative  endo-1,4-beta-xylanase (XlnA), putative  hydrophobin, putative  NRPS-like enzyme, putative  cell wall protein PhiA  endo-1,3(4)-beta-glucanase, putative  conserved hypothetical protein  O-methyltransferase, putative  glyceraldehyde 3-phosphate dehydrogenase, putative  endoglucanase, putative  monooxygenase, putative | 0.004  0.013  0.019  0.020  0.023  0.026  0.027  0.027  0.028  0.029  0.030  0.034  0.035  0.035  0.037  0.038  0.038  0.039  0.040  0.041 | 0.002  0.002  0.000  0.000  0.000  0.000  0.001  0.001  0.000  0.001  0.001  0.000  0.003  0.002  0.008  0.007  0.002  0.001  0.001  0.002 | 3.091  4.941  3.946  9.034  5.896  3.198  1.866  1.100  7.000  6.159  2.052  9.749  3.297  4.637  4.768  1.445  5.632  4.741  5.855  4.678 |
