# Supplementary material for: The Putative APSES Transcription Factor RgdA Governs Growth, Development, Toxigenesis, and Virulence in Aspergillus fumigatus
Source: mSphere. 2020 Nov 11;5(6):e00998-20. doi: 10.1128/mSphere.00998-20 (PMC7657592; doi:10.1128/mSphere.00998-20)
Supplement: TABLE S3 [file mSphere.00998-20-st003.docx]

**TABLE S3** Oligonucleotides used in this study^a^

| Name | Sequence (5'→3') | Purpose |
| --- | --- | --- |
| oligo697  oligo698  oligo374  oligo375  oligo376  oligo377  oligo378  oligo379  oligo699  oligo700  oligo256  oligo257  oligo303  oligo304  oligo305  oligo306  oligo689  oligo690  oligo691  oligo692  oligo693  oligo694  oligo1008  oligo1009  oligo1264  oligo1265  oligo1084  oligo1085  oligo1297  oligo1298  oligo1299  oligo1300  oligo1301  oligo1302  oligo258  oligo259  oligo269  oligo270  oligo1274  oligo1275  oligo430  oligo431  oligo434  oligo435  oligo1295  oligo1296  oligo508  oligo509  oligo514  oligo515  oligo1310  oligo1311  oligo1312  oligo1313  oligo1376  oligo1377  oligo1515  oligo1516 | GCAATGTAAAGCTAACGTGCGTG  TGCCTTTAAGCTTCGGGTAGAG  CTGCCGAAAAACCGAAATTA  *TTTGTAGGCTTTGGGCTGTTCACAA*AAAGAGGCATTGGGGAAAAT  *CTGATCTACCCCTTGGAACGCAGCA*GTAACTTTGGCTGGGAAGCA  CGTCTTCAAGTGGCCTTGTT  TCTTGACGGGAAGCGACTAT  CCACTTGCAGGATGTGTGAT  CCATGTGTGTCGAGTCCTTC  GAACGTACAGCAACAGTCTGG  TTCCAAGCAGAGCTTGTCAC  CCAGGTTCTTTGCACTTGAA  GCTACCACTCTGCATCCTCA  TACGAGCTCCAGCATGATTC  ACGGCAGGAAGTTGTCTTCT  CTGTCAGCGACTTGTTGGAT  AAACCCCTGTGAATGCAGAC  CCCCTTGAGATGAAAGGTGA  CGATCTGTACCCCAACGAGT  TTCTGGAACTTTGCCAGCTT  ACTCCACCATCCAGTTCCAG  TCCGAGTATCCCTCGATGTC  TCACGGCATACAGCGACTAT  AAGAAGACCTCCCACCTTGG  ACGGTGGCGGATATCGGGTGGTA  AAGACGAGGACGGCGGCGAGAAAG  ATGGCGGCGGTGGATTTCTC  CCTCGTCGGCGCATAACACTTT  GGCTACGGCACCACTTCCTCA  CGGTTGTCCTGGTCGCTGTCC  CGGGGCCCAGACGGTGAAAAG  GTGAGGGCCGTGAGTGGTATTGGT  CACCGACCCGTTTACTGGACCTG  GACGCGGGGATCGAGAGTGTTGG  CTCTTCAACCAGTGCTCCAA  GCAATGTTCTGCTTGCACTT  TGAAGTTCCTCGCTGTTGTC  GTGTTCACCGCACTTGTTCT  GATGCGCAAGTCCCTGTGGTC  ACGCATCGCCGGGGTTCTTCT  TTCCAAATGTGGCAAGTGAT  GCAAACGTGGAATCAATACG  CCACCACCTACAACAACAGC  TGTGAAGACGCATGATGAGA  TGCGCGGCGATTTAACGAGACTA  CGGCTTCAGATCACGGTAAAC  GAGAATACCCTGCGGTTTGT  GGATCCTCTTCTTGGGATCA  ACTCCGAGGATGAGGATGAC  GACCCTGTTCCTTTCCAAGA  GGGCAAGAATCGGTCGCAGAAGTT  CGCAGGGATGGCACCGAAGTAAGA  CGCGGCGATACTTCCAGGTT  CATAATCGCCCACTCGTTGTCTA  CGATCGGGCGCAAACTCACG  TCCCCCTCCCGCGATTGTAACTTC  *TAGTTCTGTTACCGAGCCGG*CTTCCCAGCCAAAGTTAC  *GCTCTGAACGATATGCTCCAAC*CATGTCCATATGAGTGAACCC | 5' *AnipyrG* marker  3' *AnipyrG* marker  5' flanking region of *rgdA*  5' *rgdA* with *AnipyrG* tail  3' *rgdA* with *AnipyrG* tail  3' flanking region of *rgdA*  5' nested of *rgdA*  3' nested of *rgdA*  5' *ef1α* for qRT-PCR normalization  3' *ef1α* for qRT-PCR normalization  5' *brlA* for qRT-PCR  3' *brlA* for qRT-PCR  5' *abaA* for qRT-PCR  3' *abaA* for qRT-PCR  5' *wetA* for qRT-PCR  3' *wetA* for qRT-PCR  5' *gliP* for qRT-PCR  3' *gliP* for qRT-PCR  5' *gliM* for qRT-PCR  3' *gliM* for qRT-PCR  5' *gliT* for qRT-PCR  3' *gliT* for qRT-PCR  5' *gliK* for qRT-PCR  3' *gliK* for qRT-PCR  5' *gliA* for qRT-PCR  3' *gliA* for qRT-PCR  5' *rgdA* for qRT-PCR  3' *rgdA* for qRT-PCR  5' *dprA* for qRT-PCR  3' *dprA* for qRT-PCR  5' *dprB* for qRT-PCR  3' *dprB* for qRT-PCR  5' *dprC* for qRT-PCR  3' *dprC* for qRT-PCR  5' *rodA* for qRT-PCR  3' *rodA* for qRT-PCR  5' *rodB* for qRT-PCR  3' *rodB* for qRT-PCR  5' *rodC* for qRT-PCR  3' *rodC* for qRT-PCR  5' *acyA* for qRT-PCR  3' *acyA* for qRT-PCR  5' *pkaC1* for qRT-PCR  3' *pkaC1* for qRT-PCR  5' *pkaC2* for qRT-PCR  3' *pkaC2* for qRT-PCR  5' *sakA* for qRT-PCR  3' *sakA* for qRT-PCR  5' *atfA* for qRT-PCR  3' *atfA* for qRT-PCR  5' *pbs2* for qRT-PCR  3' *pbs2* for qRT-PCR  5' *sskB* for qRT-PCR  3' *sskB* for qRT-PCR  5' *ssk1* for qRT-PCR  3' *ssk1* for qRT-PCR  5' *rgdA* with *hygB* tail for complementation  3' *rgdA* with *hyg*B tail for complementation |

^a^Tail sequence is in italic.
